# Team-based primary care reforms and older adults: a descriptive assessment of sociodemographic trends and prescribing endpoints in two Canadian provinces

**DOI:** 10.1186/s12875-022-01960-z

**Published:** 2023-01-10

**Authors:** Nichole Austin, David Rudoler, Sara Allin, Lisa Dolovich, Richard H. Glazier, Agnes Grudniewicz, Elisabeth Martin, Caroline Sirois, Erin Strumpf

**Affiliations:** 1 Dalhousie University, Halifax, Canada; 2 Ontario Tech University, Oshawa, Canada; 3 Institute for Clinical and Evaluative Sciences, Toronto, Canada; 4 Ontario Shores Centre for Mental Health Sciences, Whitby, Canada; 5 University of Toronto, Toronto, Canada; 6 St. Michael’s Hospital, Toronto, Canada; 7 University of Ottawa, Ottawa, Canada; 8 Université Laval, Quebec City, Canada; 9 McGill University, Montreal, Canada

**Keywords:** Primary care, Team-based care, Older adults, Canada, Polypharmacy, Medication management

## Abstract

**Background:**

Team-based primary care reforms aim to improve care coordination by involving multiple interdisciplinary health professionals in patient care. Team-based primary care may support improved medication management for older adults with polypharmacy and multiple points of contact with the healthcare system. However, little is known about this association. This study compares sociodemographic and prescribing trends among older adults in team-based vs. traditional primary care models in Ontario and Quebec.

**Methods:**

We constructed two provincial cohorts using population-level health administrative data from 2006–2018. Our primary exposure was enrollment in a team-based model of care. Key endpoints included adverse drug events (ADEs), potentially inappropriate prescriptions (PIPs), and polypharmacy. We plotted prescribing trends across the observation period (stratified by model of care) in each province. We used standardized mean differences to compare characteristics of older adults and providers, as well as prescribing endpoints.

**Results:**

Formal patient/physician enrollment increased in both provinces since the time of policy implementation; team-based enrollment among older adults was higher in Quebec (47%) than Ontario (33%) by the end of our observation period. The distribution of sociodemographic characteristics was reasonably comparable between team-based and non-team-based patients in both provinces, aside from a persistently higher share of rural patients in team-based care. Most PIPs assessed either declined or remained relatively steady over time, regardless of model of care and province. Several PIPs were more common among team-based patients than non-team-based patients, particularly in Quebec. We did not detect notable trends in ADEs or polypharmacy in either province.

**Conclusions:**

Our findings offer encouraging evidence that many PIPs are declining over time in this population, regardless of patients’ enrollment in team-based care. Rates of decline appear similar across models of care, suggesting these models may not meaningfully influence prescribing endpoints. Additional efforts are needed to understand the impact of team-based care among older adults and improve primary care prescribing practices.

**Supplementary Information:**

The online version contains supplementary material available at 10.1186/s12875-022-01960-z.

## Background

The proportion of people aged 65 and over has nearly doubled across OECD countries since 1960 [1]. This trend is reflected in Canada, where the population of older adults is expanding faster than any other age demographic [2]. While increasing longevity is not necessarily synonymous with poor health [3], older adults constitute the largest share of so-called “high-cost” healthcare users [4] and many have complex health needs. It is consequently common for older adults to have multiple points of contact with the healthcare system. Many are also frequent users of prescription medications, leading to widespread concerns about medication management and associated adverse events in this population [5–7]. Most Canadian older adults use five or more distinct drug classes annually [8], but quality is more important than quantity: over 30% use at least one potentially inappropriate prescription (PIP) [9, 10], which can lead to poor individual outcomes (e.g., hospitalization due to adverse drug events (ADEs)) and increased costs to health care systems [9–17].

Team-based primary care is one approach that may promote healthy aging by improving the coordination and application of expertise across disciplines providing patient care for older adults [18, 19], which could translate to improved medication management. In Canada, primary care is considered medically necessary and is therefore covered by the public health insurer, with provision managed by each of the provincial/territorial governments [20, 21]. Accordingly, primary care strategies vary by province. Ontario and Quebec, Canada’s most populous provinces, introduced team-based primary care reforms in the early 2000s. Ontario’s Family Health Teams (FHTs), introduced in 2005 [22, 23], include a mix of family physicians and interdisciplinary providers such as nurse practitioners, registered nurses, pharmacists, social workers, and others. As of 2016, FHTs served approximately 25% of Ontarians [20, 24]. In Quebec, Groupes de médecine de famille (GMFs) were introduced in late 2002 and include family physicians, nurses, and (in recent years) other health care providers [25]. While early GMFs were more common in rural settings, these practices are now a common model of care throughout the province, with about 60% of the population enrolled in a GMF [26].

Team-based models are designed to offer a consolidated and coordinated point of care for patients, which could ultimately streamline medication review and prescribing practices (particularly given selected provincial efforts to support medication management [20]). However, evidence on this relationship is currently lacking: while some findings suggest that team-based models reduce emergency department use and improve processes of care for individuals with certain conditions [22, 27], it is unclear if these improvements are partially driven by improved medication coordination and management. Furthermore, evidence on the impact of FHTs/GMFs on use of medications specifically among older adults remains sparse, due in large part to the complexity of the patient population and the dynamic nature of patient enrollment with family physicians. In both provinces, providers are free to opt into team-based models, and patients are likewise free to choose their primary care provider (although these decisions are ultimately constrained by provider availability) [21]. As a result, the same characteristics that might encourage a provider to join a team-based practice (or encourage an older adult to seek out/enroll with a team-based provider) may also influence the probability of adverse medication-related events. There is also potential heterogeneity between provinces, particularly given differences in team composition (for example, the presence of a pharmacist on the team) [20].

It would be informative for policy makers considering primary care model options to understand if older adults in team-based care – and their providers – differ from those who are not in team-based care. An analysis of early adopters in Quebec [25] reported systematic differences between GMF-enrolled patients/physicians and their non-GMF counterparts, but it is unknown if such differences persisted as team-based practice became the dominant primary care model in that province. There are also systematic differences between people enrolled in FHTs and those who are not [27]. No analyses to-date have assessed the evolution of team-based (vs. non-team-based) older adults’ characteristics and trends in medication management.

Our aim was to describe the characteristics of older adults receiving care in team-based (vs. non-team based) practices in Ontario and Quebec, paying particular attention to within- and between-province temporal trends in sociodemographic composition. We also assess several common indicators of medication management in this population, exploring trends over time and by province/model of care, as well as provider characteristics.

## Methods

We constructed two open cohorts of older adults using population-level health administrative data in Quebec and Ontario, with exclusion criteria and relevant measures harmonized across provinces.

In Quebec, we partnered with the Institut national d’excellence en santé et en services sociaux (INESSS) to construct our cohort [28]. In Ontario, comparable data were requested through and compiled by ICES (formerly known as the Institute for Clinical Evaluative Sciences). Databases accessed for this study included: registry files from provincial regulatory colleges, physician billing information, hospital separation and emergency department visit data, prescription drug claims, and patient registration files for provincial insurers. In Ontario, these datasets were linked using unique encoded identifiers and analyzed at ICES. ICES is an independent, non-profit research institute whose legal status under Ontario’s health information privacy law allows it to collect and analyze health care and demographic data, without consent, for health system evaluation and improvement. Study approval was granted by the Research Ethics Board (REB) at Ontario Tech University (file number 14877). An expedited approval was issued by the McGill University Institutional Review Board (IRB) for work conducted in Quebec, as this study involved no more than minimal risk. All data were anonymized.

We assessed our cohorts across a series of repeated cross-sections to examine changes over time. To ensure an adequate number of team-based practices in each year, we focused our analysis on fiscal years 2006–2018. Although several GMFs existed in Quebec prior to the start of this observation period, these practices were unique and served a higher-morbidity (and therefore less representative) population [25, 29]. Eligible patients were between 66 and 104 years of age (inclusive) at the beginning of each fiscal year (April 1st), had valid data on sex and date of birth, were not in long-term care, and were covered by the public provincial insurers (the Ontario Health Insurance Plan (OHIP) or the Régie de l’assurance maladie du Québec (RAMQ)) for at least ¾ of the preceding fiscal year. The length of follow-up varied by patient (ranging anywhere from one year to the full observation period), and was determined by both the year of cohort entry as well as an annual reassessment of eligibility (for example, an otherwise eligible patient who transitioned to long-term care in 2010 would only be included in our study up to that point). We captured a wide range of sociodemographic and service-related data on patients in both provinces, including patients’ age, sex, rurality, area-level socioeconomic characteristics, number of unique prescribers/prescriptions, and patient/physician interactions per year. We also captured relevant physician-level data (sex, years in practice, average number of older adult patients per physician).

Patients were considered “exposed” to FHT/GMF models based on the affiliation of their family physician in each fiscal year. Our patient/physician linkage strategy joined patients to the family physician with whom they were formally enrolled for at least ¾ of the fiscal year (if applicable). We also constructed a second linkage based on patients’ usual provider of care, or UPC (the family physician with whom a given patient had the maximum number of patient/day interactions in a given fiscal year) to examine the robustness of our findings to an alternative definition of patient/provider attachment. As findings were consistently robust to the choice of linkage/attachment definition, we rely on the “formal enrollment” definition throughout this analysis.

Patient-level endpoints included three common indicators of medication management in older adults: adverse drug events (ADEs), potentially inappropriate prescriptions (PIPs), and polypharmacy. These endpoints are important and informative indicators of overall medication use and medication appropriateness/related complications in this population [5–10, 20]. ADEs were defined based on ICD-10 codes maintained by the Canadian Institute for Health Information (CIHI) reflecting adverse drug reaction–related hospitalizations among older adults (Additional file 1: Appendix 1). We also derived a basic indicator of polypharmacy, which we describe in greater detail below. Measures of polypharmacy do not reliably reflect medication appropriateness: for example, a person with a complex condition may require several (appropriate) medications simultaneously. Nevertheless, polypharmacy is a commonly used measure of medication management, so we include it as a secondary outcome. As nearly all Quebec adults (65+) and all Ontario older adults (65+) are covered publicly for prescription drug costs [30–33], our data contain most prescription drug dispensations in the two provinces.

The aim of this analysis was to descriptively compare patient/physician characteristics and prescribing trends across the two provinces, and across models of care (team-based vs. non-team-based). We began by harmonizing data across Ontario and Quebec. Challenges emerged in defining our outcome measures given differences in the provincial data holdings: for example, algorithms to create Beer’s List and STOPP/START criteria indicators, two commonly used measures of potentially inappropriate prescribing [34, 35], were available in Ontario but not Quebec (and not easily transportable from one context to the other). To build common measures of PIPs across provinces in the absence of these validated measures in the Quebec data, the pharmacists on our team conducted an extensive and iterative review of distinct drugs (and combinations of drugs) that are almost always contraindicated among older patients. This expert-driven, consensus-based process yielded the list of drugs and combinations in Table 1. For the joint prescribing outcomes, we required the prescriptions to overlap for at least 14 days (based on a combination of the dispensation date and the duration of the prescriptions).

**Table 1:** Specific drug classes & combinations that represent PIPs

Individual drugs	Combinations
Opioids	Opioids + benzodiazepines
Benzodiazepines	NSAIDs + antiplatelets (without PPI)
Proton pump inhibitors (PPI)	NSAIDs + anticoagulants
Anticholinergics	
Long-acting sulfonylureas	

*PIPs* Potentially inappropriate prescriptions, *NSAIDs* Nonsteroidal anti-inflammatory drugs, *PPI* Proton pump inhibitor

Another challenge was that was that Ontario and Quebec rely on different drug classification systems (Anatomical Therapeutic Chemical (ATC) and American Hospital Formulary Service (AHFS) classification systems, respectively), which initially prevented a comparable count of unique medications between provinces, and therefore a comparable measure of polypharmacy. To resolve this issue, we used a crosswalk file in Quebec to map drug identification numbers (DINs), available in both provinces, to ATC codes, and constructed a measure of polypharmacy defined as five or more different drug classes (3rd level ATC) per fiscal year. This approach, which has been employed in other work [36, 37] yields a simple measure that is consistent across provinces.

We summarized patient/physician characteristics and prescribing measures across repeated cross-sections of our observation period. We also plotted prescribing trends across the observation period, stratified by FHT/GMF status, in each province.

## Results

**Fig. 1:**
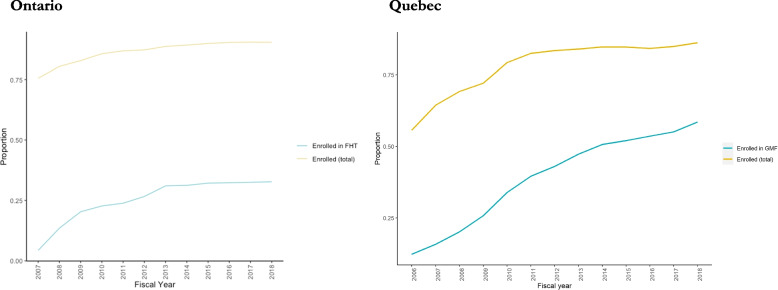
Trends in FHT/GMF enrollment among older adults

Figure 1 illustrates trends in enrollment with a family physician among older adults in Ontario and Quebec. Formal patient/physician enrollment (overall and with an FHT/GMF physician in particular) increased in both provinces since the time of policy implementation, as expected. In Quebec, while the proportion of older adults in our cohort who were enrolled with any family physician began to plateau at 87%, the proportion enrolled with a GMF physician continued to climb, reaching 47% in 2018. In Ontario, the share of older adults enrolled with a family physician plateaued at roughly 90%, but the share enrolled in FHTs had not surpassed 33%. Most older adults in our cohort – approximately 65% in both provinces as of 2018 - were enrolled with the physician who was also their usual provider of care; in other words, patients in our cohort were likely to receive the majority of their care from the provider with whom they were enrolled. This proportion increased over our observation period (Additional file 1: Appendix 2).

Tables 2 and 3 summarize the evolution of relevant patient and physician characteristics over time. These tables are stratified by FHT/GMF status at each point in time (note that patients may migrate between groups over time). In both provinces, aside from a persistently higher share of rural patients in team-based care, the distribution of sociodemographic characteristics was reasonably comparable between these two groups, particularly in the later years when FHTs/GMFs were better established (FYs 2010–18). FHT/GMF physicians were more likely to be female (particularly in Quebec) with fewer years in practice. GMF physicians had fewer enrolled older patients than non-GMF physicians, while FHT physicians had more enrolled older patients than non-FHT physicians.

**Table 2:** Patient and provider characteristics over time: FHT vs. non-FHT

	ON							
	FHT				Non-FHT			
	2006	2010	2014	2018	2006	2010	2014	2018
**Patient characteristics**								
N	59,236	322,190	444,980	519,463	1,486,582	1,394,286	1,529,972	1,682,341
Age (mean (sd))	75.7 (6.9)	75.8 (7.3)	75.5 (7.5)	75.5 (7.5)	75.6 (7.1)	75.7 (7.3)	75.5 (7.5)	75.5 (7.6)
Female	57.4%	56.6%	55.7%	55.3%	56.6%	55.8%	55.0%	54.6%
Rural	20.7%	22.3%	23.0%	22.3%	13.7%	11.9%	9.8%	9.6%
Unique prescribers (mean (sd))	2.5 (1.8)	2.7 (1.9)	3.3 (2.3)	3.3 (2.3)	2.3 (1.7)	2.5 (1.9)	3.0 (2.3)	3.0 (2.3)
Unique drugs prescribed (mean (sd))^a^	7.5 (5.4)	7.9 (5.7)	8.0 (5.8)	7.8 (5.7)	7.5 (5.6)	8.0 (6.0)	8.0 (6.1)	7.7 (6.0)
Primary care visits (mean (sd))	6.2 (8.1))	4.6 (5.6)	4.3 (5.3)	4.1 (5.1)	8.4 (9.8)	5.4 (6.7)	4.9 (6.2)	4.7 (5.9)
Income quintile								
Missing	0.42%	0.4%	0.2%	0.2%	0.3%	0.3%	0.3%	0.3%
1 (highest)	15.8%	21.3%	19.8%	20.6%	19.9%	20.4%	19.2%	19.4%
2	17.6%	20.5%	18.7%	19.3%	19.3%	19.9%	18.1%	18.5%
3	20.3%	19.4%	20.2%	20.1%	19.3%	19.6%	19.7%	19.9%
4	24.1%	19.8%	21.2%	20.5%	21.0%	20.7%	21.6%	21.3%
5 (lowest)	21.8%	18.6%	20.1%	19.4%	20.2%	19.2%	21.1%	20.6%
**Provider characteristics**^b^								
N	211	1,680	2,614	2,886	10,902	10,182	11,973	12,041
Years in practice (mean (sd))	22.9 (9.2)	22.9 (11.0)	22.8 (12.2)	22.2 (12.6)	23.3 (12.3)	24.7 (13.0)	25.1 (14.7)	23.9 (14.6)
Female	38.86%	42.86%	45.64%	49.24%	36.15%	39.65%	43.24%	46.86%
Mean older (66+) patients per physician^c^ (sd)	208.6	243.2	303.2	335.5	171.7	183.9	216.0	275.2

^a^Unique drugs defined according to ATC code (3rd level). ^b^Captures only providers who interact with patients in our sample (not illustrative of all ON family physicians). Likewise, statistics on the number of patients per physician reflects only patients in our sample (these numbers would increase substantially if we were to look at the general population)

**Table 3:** Patient and provider characteristics over time: GMF vs. non-GMF

	QC							
	GMF				Non-GMF			
	2006	2010	2014	2018	2006	2010	2014	2018
**Patient characteristics**								
N	110,434	339,807	587,022	784,561	785,594	663,080	571,228	556,711
Age (mean (sd))	74.9 (6.5)	75.0 (6.8)	74.9 (7.0)	74.8 (7.0)	74.8 (6.6)	75.0 (6.9)	74.9 (7.2)	74.9 (7.2)
Female	58.9%	57.4%	56.4%	55.2%	58.0%	56.9%	55.4%	54.7%
Rural	28.1%	26.1%	25.9%	24.1%	20.6%	19.1%	17.3%	18.5%
Unique prescribers (mean (sd))	4.9 (2.2)	5.4 (2.6)	5.7 (2.7)	5.6 (2.5)	4.8 (2.2)	5.3 (2.6)	5.5 (2.7)	5.4 (2.5)
Unique drugs prescribed (mean (sd))^a^	6.6 (4.2)	6.8 (4.5)	7.0 (4.6)	6.9 (4.7)	6.0 (4.3)	6.3 (4.6)	6.4 (4.8)	6.4 (4.8)
Primary care visits (mean (sd))	6.0 (8.7)	5.6 (8.8)	5.1 (8.4)	4.6 (7.5)	6.0 (9.1)	5.5 (9.2)	5.1 (9.1)	4.7 (8.4)
Income quintile^b^								
Missing	6.1%	6.4%	2.5%	2.3%	6.3%	6.6%	2.8%	2.6%
1 (highest)	10.5%	13.0%	13.3%	15.1%	12.2%	13.1%	14.5%	15.5%
2	17.0%	18.6%	18.1%	19.3%	16.7%	17.2%	16.6%	17.0%
3	19.3%	20.3%	21.5%	21.7%	20.8%	20.7%	20.0%	20.0%
4	23.5%	21.8%	22.9%	21.8%	21.9%	21.1%	22.6%	22.2%
5 (lowest)	23.7%	19.8%	21.7%	19.9%	22.1%	21.3%	23.5%	22.7%
**Provider characteristics**^c^								
N	943	2,354	3,552	4,435	3,720	3,085	2,272	2,128
Years in practice (mean (sd))	18.9 (9.5)	20.7 (10.7)	21.6 (12.1)	20.6 (13.4)	21.2 (9.2)	23.4 (10.6)	25.2 (11.9)	24.4 (14.0)
Female	49%	51%	55%	59%	43%	46%	49%	53%
Mean older (66+) patients per physician^c^	111.7	129.9	159.7	163	186.7	168.9	193.6	199.8

^a^Unique drugs defined according to ATC code (3rd level). ^b^Area-level deprivation measures were only available in QC in 2006 and 2016. We report the 2006 index in data years 2006/10, and the 2016 index in data years 2014/18. ^c^Captures only providers who interact with patients in our sample (not illustrative of all QC family physicians). Likewise, statistics on the number of patients per physician reflects only patients in our sample (these numbers would increase substantially if we were to look at the general population)

**Fig. 2:**
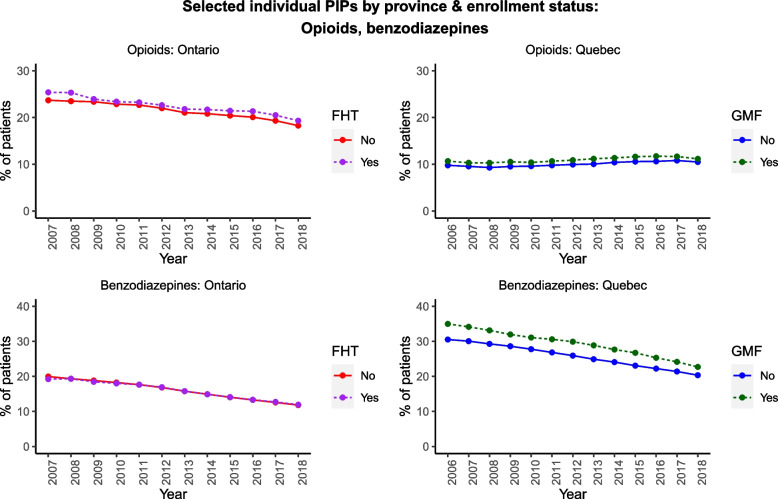
Selected individual PIPs by province/enrollment status

**Fig. 3:**
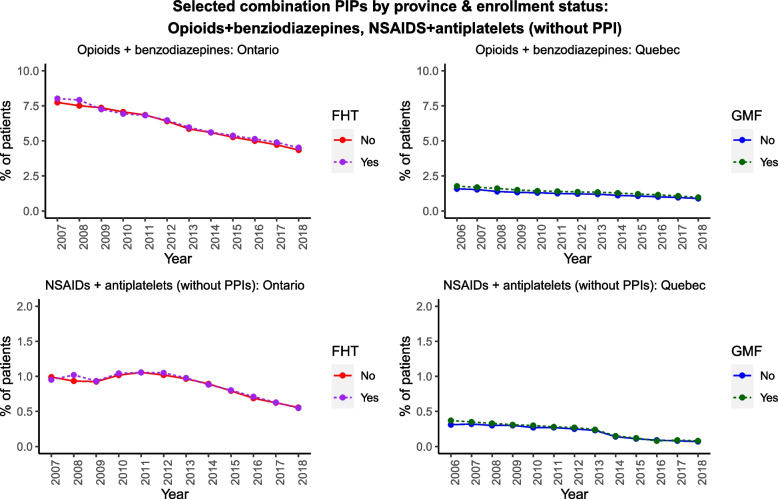
Selected combination PIPs by province/enrollment status

**Fig. 4:**
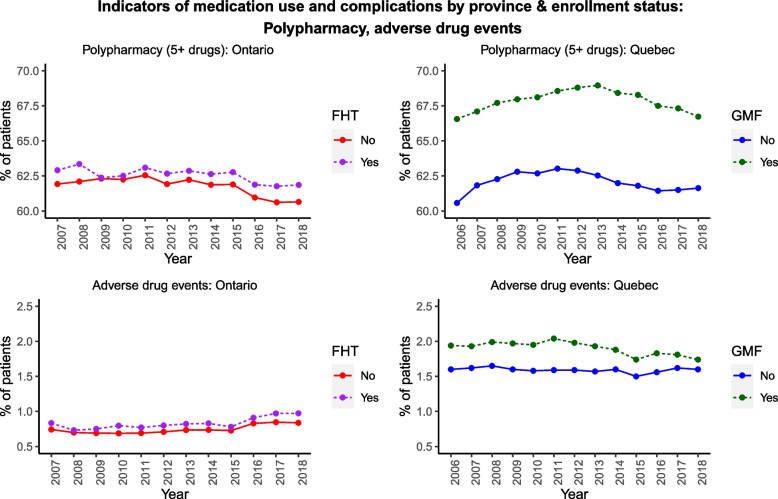
Polypharmacy (top) and adverse drug events (bottom), by province/enrollment status

Figures 2, 3, 4 illustrate selected prescribing trends by both FHT/GMF status and by province (the underlying quantitative data are tabulated in Additional file 1: Appendix 3). We focus on selected PIPs in the main text in the interest of brevity; all remaining trends are illustrated in Additional file 1: Appendix 4. Figure 2 illustrates trends in opioids and benzodiazepines. Benzodiazepine prescribing among older adults declined over time in both provinces, regardless of FHT/GMF enrollment status, and opioid prescribing declined in Ontario but remained comparatively stable in Quebec across our observation period. Most of the remaining individual PIPs (Additional file 1: Appendix 4) also declined over time in both provinces, except for PPIs.

Several individual PIPs were persistently more common among FHT/GMF patients than non-FHT/GMF patients (e.g., opioids, PPIs, anticholinergics); in contrast, long-acting sulfonylureas were less common. We also observed provincial differences in certain PIPs (e.g., benzodiazepines), which were more prevalent among team-based (vs. non-team-based) patients in Quebec, but not in Ontario. Combinations of PIPs (Fig. 3, Additional file 1: Appendix 4) were comparatively rare, but also largely declined in both provinces, regardless of enrollment status. The prevalence of these combinations was universally under 10% of our cohort in either province, with most combinations under 2%.

The percentage of patients with polypharmacy (5+ distinct drug classes) and the percentage experiencing one or more adverse drug event did not exhibit a clear trend over time in either province (Fig. 4), although polypharmacy was consistently more common among team-based (vs. non-team-based) older patients in Quebec than in Ontario. Between 60–70% of older adults were prescribed 5 or more unique drug classes each year. ADEs were much rarer (generally under 2%), regardless of FHT/GMF enrollment status.

## Discussion

Despite similar FHT/GMF (vs. non-FHT/GMF) sociodemographic profiles within each province, FHT/GMF-enrolled older adults in our sample were more commonly prescribed several of the PIPs we examined. Adverse drug events and polypharmacy were also more common among older adults enrolled in team-based care. Interestingly, the prevalence of polypharmacy remained relatively stable over our observation period even though individual and combination PIPs generally decreased over time.

As formal enrollment and FHT/GMF enrollment increased over time among older adults, so too did the preferential reliance on the primary care provider with whom the patient was enrolled, with most older patients in both provinces seeking the bulk of their primary care from the same provider. Our findings suggest that certain patient and provider characteristics – for example, patient rurality, physician gender, and the number of years a physician has been in practice – are positively associated with participation in team-based models of care in both Ontario and Quebec. However, the sociodemographic profile of FHT/GMF patients (vs. non-FHT/GMF patients) is relatively similar, and early differences between these groups have largely dissipated over time.

Most adverse endpoints were trending downward in both provinces across both team and non-team-based models of care. PPI prescribing trends were somewhat unique in this analysis as they increased while other PIPs decreased; however, this was likely due to the timing of PPI deprescribing efforts, which occurred relatively late in our observation period (we do see these trends plateau from roughly 2016 onward). The higher prevalence of certain PIPs, ADEs, and polypharmacy among FHT/GMF-enrolled older adults is potentially indicative of important underlying characteristics in this population: for example, patients with chronic conditions require more medications and may be more likely to formally enroll with a provider, which would lead to a higher incidence of (in this case, appropriate) polypharmacy among FHT/GMF enrollees. We therefore expected these trends to diverge early in the observation period (particularly in Quebec), when team-based enrollment was more common among older adults with complex health needs. However, the persistent nature of this difference suggests that, despite our findings that the sociodemographic profiles of these groups were relatively similar, FHT/GMF patients may in fact differ from their non-FHT/GMF counterparts in important ways. Future analyses should consider incorporating measures of morbidity and prescriber characteristics to better understand the observed trends in adverse prescribing outcomes. It is also important to note that our approach does not reflect the complexity of prescribing in this patient population: the physician with whom a patient is enrolled is often not their only prescriber, as illustrated by the number of “unique prescribers” in each province (Tables 2 and 3). Similarly, our data did not capture information on the other providers within each team (e.g., pharmacists, nurses), or their respective roles in medication management; while the link between a patient and their physician is important, a more comprehensive portrait of team composition would be helpful in further exploring the trends identified in this analysis.

Our results are consistent with previous work [25], despite our reliance on a broader patient cohort. Our findings reinforce existing evidence that the prescription of key PIPs (e.g., opioids, benzodiazepines) is declining [38, 39]. We also observed noteworthy provincial differences in opioid and benzodiazepine prescribing, with lower opioid prescribing in Quebec and lower benzodiazepine prescribing in Ontario; these findings align with previous work [38–41]. Finally, recent work similarly noted declines in PIPs without a corresponding change in the number of medications prescribed among older patients in team-based care (although this study was focused on the impact of pharmacist-led medication review) [42]. This could plausibly arise when providers deprescribe potentially harmful medications in favor of beneficial medications, thus reducing a patient’s PIP count while maintaining their count of unique drugs.

Our study had several limitations, many of which were linked to our prescribing measures (and associated data challenges). First, our ADE definition was based on an established list of ICD-10 codes, but this list is conservative and only captured drug events severe enough to result in hospitalization. Second, given the lack of validated measures of inappropriate prescribing (e.g., Beer’s List, STOPP/START criteria) in Quebec, and in the interest of generating a harmonized measure of inappropriate prescribing, we relied on the substantive knowledge of our team’s pharmacists to generate a list of commonly contraindicated drugs and combinations in this population. This list is not formally validated, but all the included medications appear in one or both of the Beer’s List and STOPP/START criteria. As such, our findings likely underestimate the prevalence of PIPs compared to these established criteria. Another limitation was that Ontario and Quebec rely on different drug classification systems (ATC and AHFS, respectively), which initially prevented a comparable count of unique medications between provinces, and therefore a comparable measure of polypharmacy. We resolved this issue by mapping DINs (available in both provinces) to ATC codes to build our harmonized measure of polypharmacy. It is also important to note that our prescribing data reflects dispensations (prescriptions filled and paid for by the public insurer) rather than consumption. Finally, area-level measures of deprivation were only available in Quebec in 2006 and 2016. We used the 2006 indices in the first half of our data period (FY2006–10) and the 2016 indices in the later period (FY2011–18). Some misclassification is inevitable but likely negligible given the high correlation (ranging from .7 to .8) between these area-level deprivation measures over time. Data limitations also limited our ability to describe and adjust for case mix.

## Conclusions

This descriptive work illustrates both the sociodemographic characteristics of older adults in team-based care (and their non-team-based counterparts) and trends in prescribing outcomes in two Canadian provinces. Our findings offer encouraging evidence that many PIPs are declining over time in this population, regardless of patients’ enrollment in team-based care. Rates of decline appear to be quite similar across models of care, which suggests that these models may not meaningfully influence prescribing endpoints (although additional investigation is necessary to determine if this is the case, given the inherent limitations of this descriptive analysis).

While our analysis does not quantify the impact of FHTs/GMFs on adverse prescribing endpoints in older adults, our descriptive approach offers valuable insight regarding the composition of these groups and key temporal trends, both of which are essential considerations in estimating the impact of these models of care. Our use of a population-level database allows us to present comprehensive estimates on a key demographic of interest. We also contribute an alternative framework for assessing PIPs in contexts without access to standard measures (e.g., Beer’s, STOPP/START). Finally, to our knowledge, there have been no comparative studies evaluating how different approaches to implementing team-based care across provinces have affected medication management, nor have there been any attempts to understand the mechanisms by which improvements in medication management and associated outcomes may arise in team-based care. This study is a first step toward these larger goals. Accordingly, this work is useful at face value (e.g., for providers seeking additional information on their patient populations) and as a catalyst for further rigorous evaluative analyses.

## Supplementary Information

**Additional file 1: Appendix 1.** CIHI list of ADEs. **Appendix 2.** Trends in patient/provider attachment among older adults in Ontario and Quebec. **Appendix 3.** Medication trends. **Appendix 4.** Other PIP trends, by province/enrollment status.

## Data Availability

The data that support the findings of this study are available from ICES and INESSS but restrictions apply to the availability of these data, which were used under license for the current study. These data are therefore not publicly available. Individuals interested in accessing these data may contact Nichole Austin (nichole.austin@dal.ca) for guidance on filing a formal request.

## References

[1] OECD iLibrary. Demographic trends [Internet]. Available from: https://www.oecd-ilibrary.org/sites/9989e95c-en/index.html?itemId=/content/component/9989e95c-en

[2] Statistics Canada (2016). Census Program.

[3] Lowsky DJ, Olshansky SJ, Bhattacharya J, Goldman DP (2014). Heterogeneity in healthy aging. J Gerontol Ser A: Biomed Sci Med Sci.

[4] Gray CS, Wodchis WP, Upshur R, Cott C, McKinstry B, Mercer S (2016). Supporting goal-oriented primary health care for seniors with complex care needs using mobile technology: evaluation and implementation of the health system performance research network, bridgepoint electronic patient reported outcome tool. JMIR Res Protocols.

[5] Canadian Institute for Health Information (2014). Drug Use Among Seniors on Public Drug Programs in Canada, 2012.

[6] Tamblyn RM, McLeod PJ, Abrahamowicz M, Monette J, Gayton DC, Berkson L (1994). Questionable prescribing for elderly patients in Quebec. CMAJ..

[7] Allin S, Rudoler D, Laporte A (2017). Does increased medication use among seniors increase risk of hospitalization and emergency department visits?. Health Serv Res.

[8] Canadian Institute for Health Information (2018). Drug Use Among Seniors in Canada, 2016.

[9] Morgan SG, Hunt J, Rioux J, Proulx J, Weymann D, Tannenbaum C (2016). Frequency and cost of potentially inappropriate prescribing for older adults: a cross-sectional study. Canadian Med Assoc Open Access J.

[10] Tannenbaum C, Farrell B, Shaw J, Morgan S, Trimble J, Currie JC (2017). An ecological approach to reducing potentially inappropriate medication use: Canadian Deprescribing Network. Canadian J Aging/La Revue canadienne du vieillissement.

[11] Green JL, Hawley JN, Rask KJ (2007). Is the number of prescribing physicians an independent risk factor for adverse drug events in an elderly outpatient population?. Am J Geriatr Pharmacother.

[12] Wu C, Bell CM, Wodchis WP (2012). Incidence and economic burden of adverse drug reactions among elderly patients in Ontario emergency departments: a retrospective study. Drug Saf.

[13] Armor BL, Wight AJ, Carter SM (2016). Evaluation of Adverse Drug Events and Medication Discrepancies in Transitions of Care Between Hospital Discharge and Primary Care Follow-Up. J Pharm Pract.

[14] Patel H, Bell D, Molokhia M, Srishanmuganathan J, Patel M, Car J (2007). Trends in hospital admissions for adverse drug reactions in England: analysis of national hospital episode statistics 1998--2005. BMC Pharmacol Toxicol.

[15] Lazarou J, Pomeranz BH, Corey PN (1998). Incidence of adverse drug reactions in hospitalized patients: a meta-analysis of prospective studies. JAMA..

[16] Zed PJ, Abu-Laban RB, Balen RM, Loewen PS, Hohl CM, Brubacher JR (2008). Incidence, severity and preventability of medication-related visits to the emergency department: a prospective study. CMAJ..

[17] Mekonnen AB, Redley B, de Courten B, Manias E (2021). Potentially inappropriate prescribing and its associations with health-related and system-related outcomes in hospitalised older adults: a systematic review and meta-analysis. Br J Clin Pharmacol.

[18] Starfield B, Shi L, Macinko J (2005). Contribution of primary care to health systems and health. Milbank Q.

[19] Starfield B (2009). Toward international primary care reform. Can Med Assoc J.

[20] Allin S, Martin E, Rudoler D, Carson MC, Grudniewicz A, Jopling S (2021). Comparing public policies impacting prescribing and medication management in primary care in two Canadian provinces. Health Policy.

[21] Strumpf E, Ammi M, Diop M, Fiset-Laniel J, Tousignant P (2017). The impact of team-based primary care on health care services utilization and costs: Quebec’s family medicine groups. J Health Econ.

[22] Glazier R, Kopp A, Hutchison B (2016). Comparison of family health teams to other primary care models, 2004/05 to 2011/12.

[23] Rosser WW, Colwill JM, Kasperski J, Wilson L (2011). Progress of Ontario’s family health team model: A patient-centered medical home. Ann Fam Med.

[24] Rudoler D, Peckham A, Grudniewicz A, Marchildon G (2019). Coordinating primary care services: a case of policy layering. Health Policy.

[25] Coyle N, Strumpf E, Fiset-Laniel J, Tousignant P, Roy Y (2014). Characteristics of physicians and patients who join team-based primary care practices: Evidence from Quebec’s Family Medicine Groups. Health Policy.

[26] Ministère de la santé et des services sociaux (2019). Plan stratégique 2019-2023 du Ministère de la santé et des services sociaux.

[27] Haj-Ali W, Moineddin R, Hutchison B, Wodchis WP, Glazier RH (2020). Physician group, physician and patient characteristics associated with joining interprofessional team-based primary care in Ontario, Canada. Health Policy.

[28] Austin N, Strumpf E, Rudoler D, Allin S, Sirois C, Dolovich L, et al. Regard sur l’usage des médicaments en soins et services de première ligne au Québec chez les personnes âgées inscrites à un médecin qui pratique en GMF : polypharmacie, prescription potentiellement inappropriée et effets indésirables des médicaments de 2005–2006 à 2017–2018. Québec: INESSS; 2021.

[29] Beaulieu MD, Denis JL, ’Amour D D, Goudreau J, Haggerty J, Hudon É (2006). The challenge of reorganizing practice and fostering interprofessional collaboration. Case study in five first-wave FMGs in Quebec.

[30] Gouvernement du Québec. Régime public d’assurance médicaments : modification de la participation financière de certains assures. Régie de l’assurance maladie du Québec [Internet]. Available from: https://www.ramq.gouv.qc.ca/fr/salle-presse/communiques/2020-12-29/regime-public-assurance-medicaments-modification-participation#:~:text=La%20presque%20totalit%C3%A9%20des%20personnes,plus%20grand%20nombre%20de%20m%C3%A9dicaments.

[31] Gouvernement du Québec. Statistiques de santé et de bien être selon le sexe - Tout le Québec. Ministère de la Santé et des Services sociaux du Québec [Internet]. Available from: https://www.msss.gouv.qc.ca/professionnels/statistiques-donnees-sante-bien-etre/statistiques-de-sante-et-de-bien-etre-selon-le-sexe-volet-national/participation-personnes-65-ans-et-plus-regime-public-d-assurance-medicaments/

[32] Government of Ontario. O. Reg 201:96: General. Ontario Drug Benefit Act [Internet]. Available from: https://www.ontario.ca/laws/regulation/960201

[33] Allin S, Laporte A (2011). Socioeconomic status and the use of medicines in the Ontario public drug program. Can Public Policy.

[34] Fick DM, Semla TP, Steinman M, Beizer J, Brandt N, Dombrowski R (2019). American Geriatrics Society 2019 updated AGS Beers Criteria® for potentially inappropriate medication use in older adults. J Am Geriatr Soc.

[35] Gallagher P, Ryan C, Byrne S, Kennedy J, Mahony O, D. (2008). STOPP (screening tool of older person's prescriptions) and START (screening tool to alert doctors to right treatment). Consensus validation. Int J Clin Pharmacol Therapeut.

[36] Rochon PA, Petrovic M, Cherubini A, Onder G, O'Mahony D, Sternberg SA (2021). Polypharmacy, inappropriate prescribing, and deprescribing in older people: through a sex and gender lens. Lancet Healthy Longevity.

[37] Morgan SG, Weymann D, Pratt B, Smolina K, Gladstone EJ, Raymond C (2016). Sex differences in the risk of receiving potentially inappropriate prescriptions among older adults. Age Ageing.

[38] Canadian Institute for Health Information (2019). Opioid Prescribing in Canada: How Are Practices Changing?.

[39] Gosselin E, Simard M, Lunghi C, Sirois C (2022). Trends in benzodiazepine and alternative hypnotic use in relation with multimorbidity among older adults in Quebec, Canada. Pharmacoepidemiol Drug Saf.

[40] Vogel L (2017). Seniors and self-harm factor in the opioid crisis. CMAJ..

[41] Black CD, McCarthy L, Gomes T, Mamdani M, Juurlink D, Tadrous M (2018). Interprovincial variation of psychotropic prescriptions dispensed to older Canadian adults. Canadian Geriatr J.

[42] Khera S, Abbasi M, Dabravolskaj J, Sadowski CA, Yua H, Chevalier B (2019). Appropriateness of medications in older adults living with frailty: impact of a pharmacist-led structured medication review process in primary care. J Prim Care Community Health.

