# Supplementary material for: Team-based primary care reforms and older adults: a descriptive assessment of sociodemographic trends and prescribing endpoints in two Canadian provinces
Source: BMC Prim Care. 2023 Jan 10;24:7. doi: 10.1186/s12875-022-01960-z (PMC9832790; doi:10.1186/s12875-022-01960-z)

APPENDICES

Appendix 1: CIHI list of ADEs

The ADE definition is derived from the CIHI (2013) definition:

- A most responsible diagnosis code that was either drug-related or due to a drug (see table below), provided that the most responsible diagnosis was not indicated to have occurred post-admission; or
- A pre-admission comorbidity that was either drug related or due to a drug (see table below); or
- An external cause code that was drug-related (ICD-10 codes Y40 to Y59).

Cases where the most responsible diagnosis occurred post-admission are excluded. These cases can be identified when the same diagnosis was coded as both the most responsible diagnosis and a post-admission comorbidity.

ICD-10-CA Code	Description
D52.1	Drug-induced folate deficiency anaemia
D59.0	Drug-induced autoimmune haemolytic anaemia
D59.2	Drug-induced nonautoimmune haemolytic anaemia
D61.1	Drug-induced aplastic anaemia
D64.2	Secondary sideroblastic anaemia due to drugs and toxins
D68.3	Haemorrhagic disorder due to circulating anticoagulants
D89.3	Immune reconstitution syndrome
E03.2	Hypothyroidism due to medicaments and other exogenous substances
E06.4	Drug-induced thyroiditis
E16.0	Drug-induced hypoglycaemia without coma
E23.1	Drug-induced hypopituitarism
E24.2	Drug-induced Cushing's syndrome
E27.3	Drug-induced adrenocortical insufficiency
E66.1	Drug-induced obesity
G04.0	Acute disseminated encephalitis
G21.0	Malignant neuroleptic syndrome
G21.1	Other drug-induced secondary parkinsonism
G24.0	Drug-induced dystonia
G25.1	Drug-induced tremor
G25.4	Drug-induced chorea
G25.6	Drug-induced tics and other tics of organic origin
G44.4	Drug-induced headache, not elsewhere classified
G61.1	Serum neuropathy
G62.0	Drug-induced polyneuropathy
G72.0	Drug-induced myopathy
H40.6	Glaucoma secondary to drugs
H91.0	Ototoxic hearing loss
I42.7	Cardiomyopathy due to drugs and other external agents
I95.2	Hypotension due to drugs
J70.2	Acute drug-induced interstitial lung disorders
J70.3	Chronic drug-induced interstitial lung disorders
J70.4	Drug-induced interstitial lung disorders, unspecified
K85.3	Drug-induced acute pancreatitis
L10.5	Drug-induced pemphigus
L23.3	Allergic contact dermatitis due to drugs in contact with skin
L24.4	Irritant contact dermatitis due to drugs in contact with skin
L25.1	Unspecified contact dermatitis due to drugs in contact with skin
L27.0	Generalized skin eruption due to drugs and medicaments
L43.2	Lichenoid drug reaction
L56.0	Drug phototoxic response
L56.1	Drug photoallergic response
M10.20	Drug-induced gout, multiple sites
M10.22	Drug-induced gout, upper arm
M10.24	Drug-induced gout, hand

ICD-10-CA Code	Description
M10.25	Drug-induced gout, pelvic region and thigh
M10.26	Drug-induced gout, lower leg
M10.27	Drug-induced gout, ankle and foot
M10.28	Drug-induced gout, other site
M10.29	Drug-induced gout, unspecified site
M32.0	Drug-induced systemic lupus erythematosus
M34.2	Systemic sclerosis induced by drugs and chemicals
M80.40	Drug-induced osteoporosis with pathological fracture, multiple sites
M80.42	Drug-induced osteoporosis with pathological fracture, upper arm
M80.43	Drug-induced osteoporosis with pathological fracture, forearm
M80.45	Drug-induced osteoporosis with pathological fracture, pelvic region and thigh
M80.46	Drug-induced osteoporosis with pathological fracture, lower leg
M80.48	Drug-induced osteoporosis with pathological fracture, other site
M81.4	Drug-induced osteoporosis
M83.5	Other drug-induced osteomalacia in adults
M87.11	Osteonecrosis due to drugs, shoulder region
M87.12	Osteonecrosis due to drugs, upper arm
M87.15	Osteonecrosis due to drugs, pelvic region and thigh
M87.16	Osteonecrosis due to drugs, lower leg
M87.18	Osteonecrosis due to drugs, other site
N14.0	Analgesic nephropathy
N14.1	Nephropathy induced by other drugs, medicaments and biological substances
N14.2	Nephropathy induced by unspecified drug, medicament or biological substance
R50.2	Drug-induced fever
T80.3	ABO incompatibility reaction
T80.4	Rh incompatibility reaction
T80.5	Anaphylactic shock due to serum
T80.6	Other serum reactions
T80.8	Other complications following infusion, transfusion and therapeutic injection
T80.9	Unspecified complication following infusion, transfusion and therapeutic injection
T88.1	Other complications following immunization, not elsewhere classified
T88.2	Shock due to anaesthesia
T88.3	Malignant hyperthermia due to anaesthesia
T88.5	Other complications of anaesthesia
T88.6	Anaphylactic shock due to adverse effect of correct drug or medicament properly administered
T88.7	Unspecified adverse effect of drug or medicament

Appendix 2: Trends in patient/provider attachment among older adults in Ontario and Quebec

The plots below demonstrate the nature of patient/provider attachment over time in Ontario and Quebec, respectively. Arguably, barring access barriers or suboptimal quality of care, patients should preferentially seek primary care from the family physician with whom they are enrolled in the interest of ensuring continuity of care. As of 2018 in both provinces, we found that this was the case: most older adults in our cohorts were enrolled with the physician who was also their UPC, and this proportion increased over our observation period. Less than 15% of older adults enrolled in either province routinely sought care from a different family physician. Nonusers (unenrolled individuals with no contact with a family physician most) and enrolled older adults who did not seek care from their family physician were relatively rare in both provinces over the course of the observation period. While some patients may formally disenroll from their family physician, this was quite rare in our sample, occurring in 1-2% of patients per fiscal year in Quebec.

Ontario

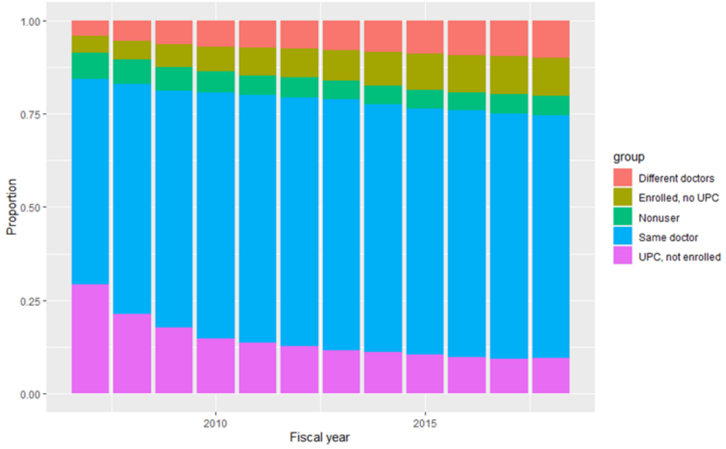

Quebec

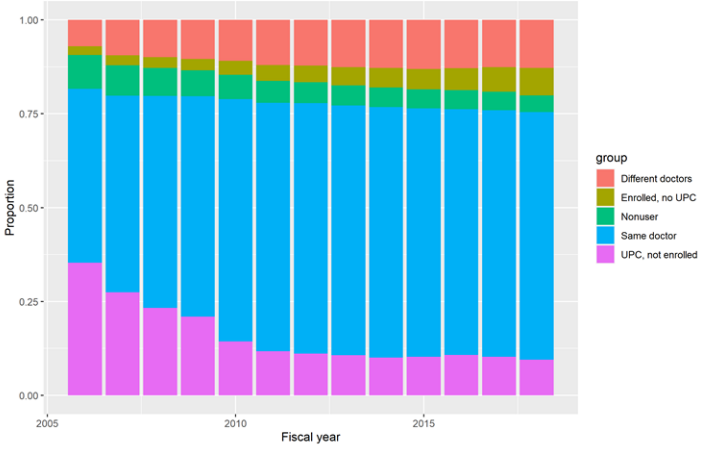

Appendix 3: Medication trends

FHT vs. non-FHT

	ON							
	FHT				Non-FHT			
	2006	2010	2014	2018	2006	2010	2014	2018
**Pharmaceutical outcomes**								
ADEs (1+)[1]	0.8%	0.8%	0.8%	1.0%	0.7%	0.7%	0.7%	0.8%
Polypharmacy[2]	62.9%	63.1%	62.8%	61.9%	61.9%	62.5%	61.9%	60.6%
PIPs[3]: specific drug classes/combinations								
Opioid (any)	25.4%	23.2%	21.4%	19.3%	23.7%	22.7%	20.4%	18.3%
Benzodiazepines	19.2%	17.6%	14.1%	11.9%	20.0%	17.6%	14.0%	11.8%
Anticholinergics	12.5%	11.6%	10.3%	9.1%	11.9%	10.7%	9.5%	8.3%
Proton pump inhibitors (PPIs)	25.5%	29.8%	31.9%	30.8%	24.0%	28.5%	30.4%	29.4%
Opioid + benzodiazepines	8.0%	6.8%	5.4%	4.5%	7.7%	6.9%	5.3%	4.3%
NSAIDs+anticoagulants	1.3%	1.3%	1.2%	1.1%	1.3%	1.2%	1.1%	1.0%
Long-acting sulfonylureas	6.0%	3.3%	1.1%	0.4%	6.8%	4.0%	1.5%	0.6%
NSAIDs + concurrent antiplatelet (without PPI)	1.0%	1.1%	0.8%	0.5%	1.0%	1.1%	0.8%	0.6%

[1]ADE=Adverse drug event (based on CIHI's list of ICD-10 codes). [2]Based conversion of DINs to 3rd level ATC codes. [3]PIP=potentially inappropriate prescription.

GMF vs. non-GMF

	QC							
	GMF				Non-GMF			
	2006	2010	2014	2018	2006	2010	2014	2018
**Pharmaceutical outcomes**								
ADEs (1+)[1]	1.9%	2.0%	1.9%	1.7%	1.6%	1.6%	1.6%	1.6%
Polypharmacy[2]	66.6%	68.1%	68.4%	66.7%	60.6%	62.7%	62.0%	61.6%
PIPs[3]: specific drug classes/combinations								
Opioid (any)	10.7%	10.4%	11.4%	11.2%	9.8%	9.6%	10.4%	10.5%
Benzodiazepines	35.0%	31.1%	27.7%	22.7%	30.5%	27.8%	24.1%	20.3%
Anticholinergics	14.0%	13.5%	13.4%	13.1%	12.5%	12.5%	12.2%	12.0%
Proton pump inhibitors (PPIs)	28.7%	33.7%	37.4%	38.3%	26.1%	30.4%	32.9%	34.4%
Opioid + benzodiazepines	1.8%	1.4%	1.3%	1.0%	1.6%	1.3%	1.1%	0.9%
NSAIDs+anticoagulants	0.3%	0.2%	0.2%	0.2%	0.3%	0.2%	0.2%	0.2%
Long-acting sulfonylureas	5.7%	3.5%	2.3%	1.8%	5.5%	3.7%	2.4%	1.9%
NSAIDs + concurrent antiplatelet (without PPI)	0.4%	0.3%	0.2%	0.1%	0.3%	0.3%	0.1%	0.1%

[1]ADE=adverse drug event (based on CIHI's list of ICD-10 codes). [2]Based conversion of DINs to 3rd level ATC codes. [3]PIP=potentially inappropriate prescription. NSAIDs: nonsteroidal anti-inflammatory drugs

Appendix 4: Other PIP trends, by province/enrollment status

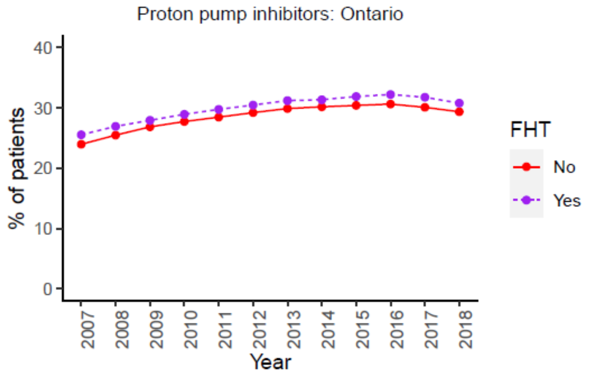

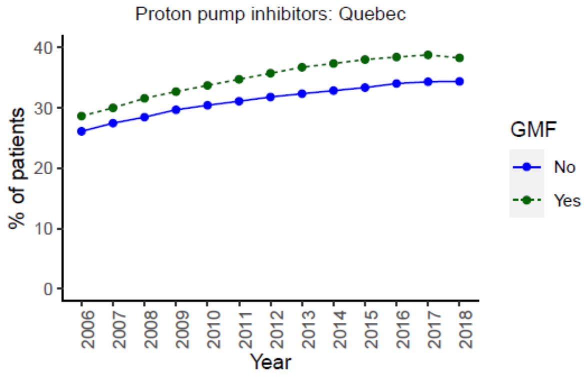

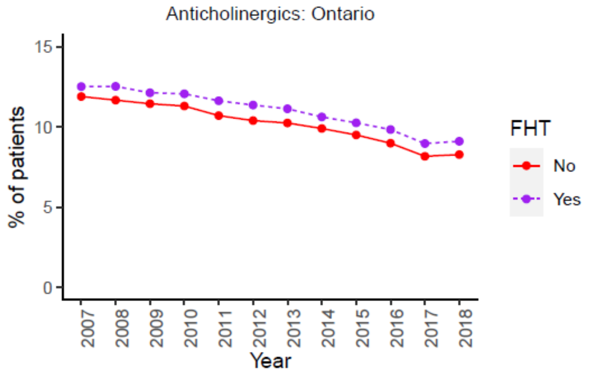

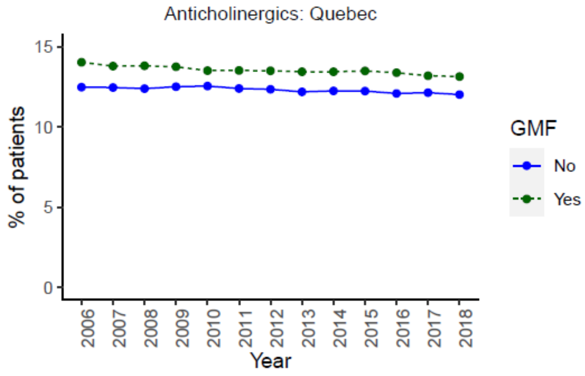

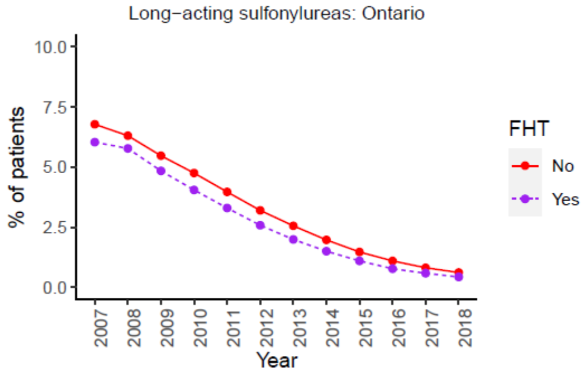

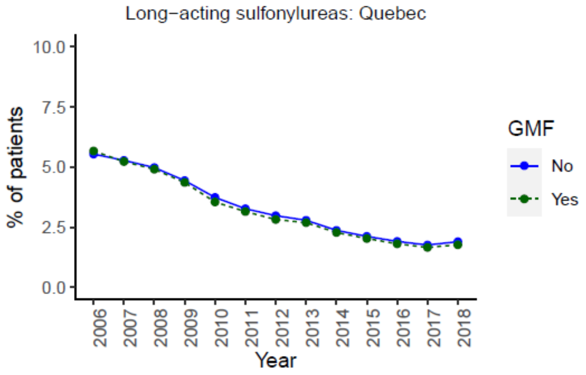

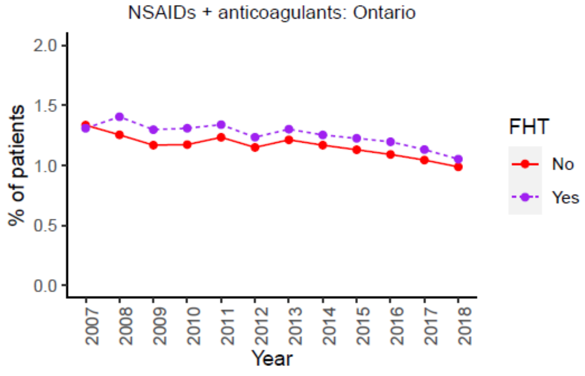

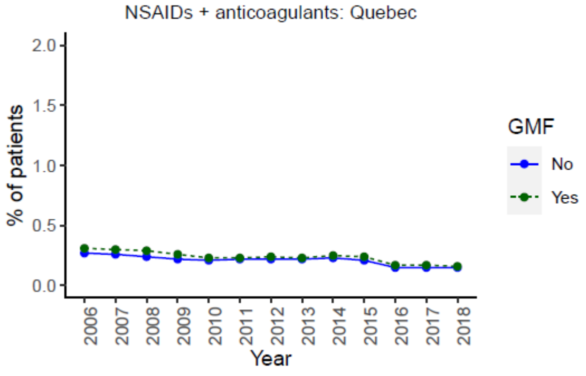

Supplement: Supplementary file 1 — Additional file 1: Appendix 1. CIHI list of ADEs. Appendix 2. Trends in patient/provider attachment among older adults in Ontario and Quebec. Appendix 3. Medication trends. Appendix 4. Other PIP trends, by province/enrollment status. [file 12875_2022_1960_MOESM1_ESM.pdf]
